# Alpha and beta phylogenetic diversities jointly reveal ant community assembly mechanisms along a tropical elevational gradient

**DOI:** 10.1038/s41598-022-11739-y

**Published:** 2022-05-11

**Authors:** Gibran Renoy Pérez-Toledo, Fabricio Villalobos, Rogerio R. Silva, Claudia E. Moreno, Marcio R. Pie, Jorge E. Valenzuela-González

**Affiliations:** 1 Instituto de Ecología, A.C. Red de Ecología Funcional, Xalapa, Veracruz Mexico; 2 Instituto de Ecología, A.C. Red de Biología Evolutiva, Xalapa, Veracruz Mexico; 3 Museu Paraense Emílio Goeldi, Coordenação de Ciências da Terra e Ecologia, Belém, PA Brazil; 4 Centro de Investigaciones Biológicas, Instituto de Ciencias Básicas e Ingeniería, Universidad Autónoma del Estado de Hidalgo, Mineral de la Reforma, Hidalgo Mexico; 5 Department of Biology, Edge Hill University, Ormskirk, Lancashire UK

**Keywords:** Biodiversity, Biogeography, Community ecology

## Abstract

Despite the long-standing interest in the organization of ant communities across elevational gradients, few studies have incorporated the evolutionary information to understand the historical processes that underlay such patterns. Through the evaluation of phylogenetic α and β-diversity, we analyzed the structure of leaf-litter ant communities along the Cofre de Perote mountain in Mexico and evaluated whether deterministic- (i.e., habitat filtering, interspecific competition) or stochastic-driven processes (i.e., dispersal limitation) were driving the observed patterns. Lowland and some highland sites showed phylogenetic clustering, whereas intermediate elevations and the highest site presented phylogenetic overdispersion. We infer that strong environmental constraints found at the bottom and the top elevations are favoring closely-related species to prevail at those elevations. Conversely, less stressful climatic conditions at intermediate elevations suggest interspecific interactions are more important in these environments. Total phylogenetic dissimilarity was driven by the turnover component, indicating that the turnover of ant species along the mountain is actually shifts of lineages adapted to particular locations resembling their ancestral niche. The greater phylogenetic dissimilarity between communities was related to greater temperature differences probably due to narrow thermal tolerances inherent to several ant lineages that evolved in more stable conditions. Our results suggest that the interplay between environmental filtering, interspecific competition and habitat specialization plays an important role in the assembly of leaf-litter ant communities along elevational gradients.

## Introduction

Elucidating the mechanisms underlying the natural variation in species richness and composition across local communities has challenged ecologists for more than a century^1^. Since the development of community phylogenetics, a vast number of studies have used the phylogenetic approach to disentangle the relative importance of both deterministic and stochastic processes involved in the coexistence of species within a community^2,3^. The cornerstone of the phylogenetic approach is the assumption of phylogenetic niche conservatism, i.e., the tendency of species to conserve their niches over evolutionary history, with closely related species being ecologically more similar to each other than to distantly related species^4^. Based on this assumption, phylogenetically clustered communities (i.e., co-occurrence of closely related species) are typically thought to result from environmental filtering, where taxa are filtered by constraints imposed by the environment^5^. However, modern coexistence theory considers that phylogenetic clustering could also be driven by competitive exclusion where entire clades have higher competitive abilities leading to an exclusion of other lineages^6^. Conversely, phylogenetically overdispersed communities (i.e., co-occurrence of distantly related species) are thought to be structured by competition, which tends to select species with different ecological traits and thus low niche overlap^7^. Finally, a lack of phylogenetic structure suggests a predominance of neutral processes^8^ or the counteraction of both habitat filtering and interspecific competition^9^. Since these mechanisms can act simultaneously within communities^10^, the critical question is no longer which mechanism structures communities but rather which one plays a dominant role in community assembly along environmental gradients^11^.

Community ecologists have expanded the community phylogenetics framework from α-(richness) to β-diversity (composition) patterns (phylogenetic beta diversity, hereafter PBD) into ecological contexts^12–14^. PBD explicitly adds phylogenetic information to evaluate how evolutionary relationships of component lineages change between communities or biomes across space^12^. Furthermore, PBD links local processes (e.g., biotic interactions or environmental filtering) to more regional evolutionary processes (e.g., trait evolution, speciation and dispersal), hence providing further information about how current or historical environmental factors influence the variation in species compositions of communities across space^12,15^. Environmental filtering and dispersal limitation have been suggested to largely determine patterns of community composition^16,17^. Habitat filtering is expected to limit community members to habitats that resemble the ancestral niche where its lineage originated, i.e., habitat specialization^18^, resulting in closely-related species occupying similar portions of regional-scale climatic gradients. On the other hand, the dispersal limitation process predicts that community dissimilarity between sites will be correlated to geographical distances separating those sites, regardless of the environmental gradients^16^. Since environmental filtering and dispersal limitation are not mutually exclusive, a greater variation in community dissimilarity predicted by geography reflects a greater importance of dispersal limitation, whereas greater variation explained by environmental distances indicates that habitat filtering is the structuring force^19^.

Recent methodological advances have improved our understanding of the origin and maintenance of geographic patterns of PBD through its decomposition into two antithetic components that account for the replacement of lineages between sites (the turnover-resultant component) and differences in the phylogenetic diversity between nested assemblages (the nestedness-resultant component^20,21^). Although the turnover and nestedness components both contribute to total dissimilarity, their relative importance depends on the processes structuring communities. For instance, if environmental conditions vary across space, and species are adapted to particular conditions, the turnover component of PBD is more likely to shape community composition under environmental filtering. Conversely, nestedness tends to be a more common component due to several processes such as selective colonization, selective extinction, nestedness of habitats or passive sampling^21^. Analyzing the relationship between PBD (and its components) with climatic and geographic variables can provide a better understanding of community assembly along environmental gradients^22^.

Several studies have placed the phylogenetic α- and β-diversity into ecological and biogeographic contexts, such as the latitudinal pattern of phylogenetic diversity^23^, the phylogenetic modification of native communities under species invasions^24^ and the community structure along elevational gradients^25–27^. Particularly, ecological gradients occurring in mountains have successfully served to test community assembly processes considering species distributions are strongly affected by the environmental conditions found at particular elevations^28,29^. Particularly for thermophilic taxa (i.e., organisms with high-temperature affinity), such as ants, deterministic processes related to both temperature^30–32^ and precipitation^26,33^ have been shown to influence community membership at elevational gradients. In tropical mountains, as elevation increases, temperature tends to decrease whereas precipitation increases^34^ and, as a result, the abiotic conditions (cold and wet) at high-elevation sites become physiologically stressful for a majority of ant species in comparison with more favorable climatic conditions (warm and humid) found at low elevations. This spatial structure created by the interplay of temperature and precipitation has been used to explain the changing phylogenetic structure from overdispersed ant communities at low elevations towards clustered communities at higher elevations^25–27^. Despite these findings, the relative importance of deterministic and stochastic processes driving ant community organization under the phylogenetic perspective is still incipient, particularly when tropical regions are considered.

In this study, we measured the phylogenetic α- and β-diversity to infer whether deterministic and stochastic processes are driving the organization of leaf-litter ant communities occurring along a tropical elevational gradient in Mexico. Leaf-litter ants have numerous attributes that make them an ideal system to explore assembly mechanisms. For instance, they exhibit high levels of local co-occurrence; with up to 35 species co-existing in only one square meter^35^. Further, ant distributions are highly constrained by local and regional climate^36,37^. They display a wide variety of both individual^38^ and colony-level^39^ thermoregulatory strategies to cope with cold and hot conditions. Lastly, molecular phylogenetic analyses of the major ant lineages are providing a stable framework to understand the evolutionary relationships of the group^40–42^. Hinged on the assumption that ant species have retained their traits along their evolutionary history (i.e., phylogenetic niche conservatism^23,43^, we expect that (1) ant communities inhabiting in favorable and stable habitats (warm and humid habitats) found at low elevations will show phylogenetic overdispersion since negative interspecific interactions might be more intense in these environments. Conversely, cold and wet conditions at high elevations would lead to phylogenetically clustered communities considering that only closely related species of a subset of lineages possess the physiological traits that allow them to persist in the harsh conditions present at those elevations. In terms of PBD, it is expected that (2) lineages would have high habitat specialization to the conditions where they originated and thus drastic environmental changes along the elevational gradient would lead to high lineage replacement. This will result in a greater importance of turnover on total phylogenetic dissimilarity along the elevational gradient. Finally, we can expect that (3) pairwise dissimilarity values for PBD and their components would be mainly explained by environmental filtering (i.e., climatic distances), following their ancestral climatic affinities rather than dispersal limitation.

## Results

### α-Diversity

**Figure 1:**
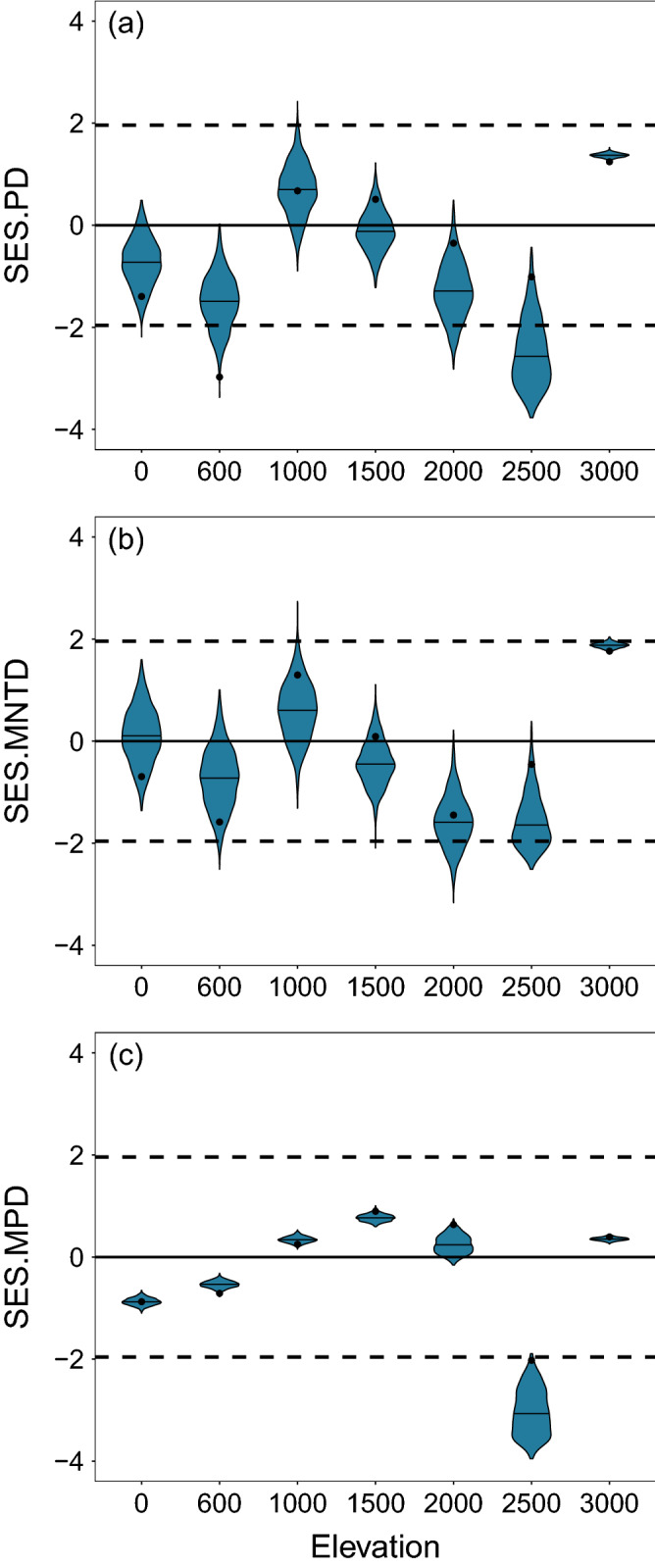
Standardized effect sizes of (**a**) phylogenetic diversity, PD; (**b**) mean nearest taxon distance, MNTD; and (**c**) mean paiwise distance, MPD of the leaf-litter ant assemblages at seven elevations along the Cofre de Perote mountain. The inner horizontal line in each violin represents the mean of the 1000 SES values per elevation. Black point represents the SES values from the MCC tree. Area of no significance is delimited by the horizontal dashed lines at 1.96 and − 1.96.

Overall, the tendency of the standardized effect sizes of phylogenetic diversity (SES.PD), mean-pairwise distance (SES.MPD) and mean nearest taxon distance (SES.MNTD) was negative values at low and some high elevational sites, whereas positive values at mid-elevations and the highest site. Considering the SES.PD, this means that three intermediate-elevational sites (1000, 1500 and 3000) contain higher evolutionary diversity in comparison with the rest of the sites (0, 600, 2000, 2500; Fig. 1a). This same trend was observed for the SES.MNTD (Fig. 1b), indicating phylogenetically diverse sites are related to more distantly species (overdispersion), whereas sites with lower phylogenetic diversity are inhabited by more closely-related species (clustering). For the SES.MPD, three mid-elevation sites (1000, 1500, 2000) and the highest site (3000) showed a tendency to overdispersion, whilst the rest of the sites (0, 600 and 2500) tended to a clustering pattern (Fig. 1c). All those trends were consistent across the 1000 phylogenetic trees and remained the same when the Maximum Clade Credibility (MCC tree) was used (Fig. 1; summary statistics for all metrics in Supplementary Table S1). Nevertheless, only the patterns at 2500 m for SES.PD and SES.MPD were significantly different from a null expectation at α = 0.05 (Supplementary Table S2).

When we assessed the contribution of climatic variables on explaining the alpha metrics patterns, the full model (Temp + Prec + Temp:Prec) explained a larger amount of variation in comparison with the additive or null model (Table 1; Supplementary Fig. S1). Moreover, the full model was considered the best statistically supported model in explaining SES.PD, SES.MPD and SES.MNTD patterns. However, in some instances, the additive model of temperature and precipitation resulted as equally probable models (Table 1, Supplementary Fig. S2), suggesting that the main effect of both variables is biologically important, yet its interaction effect refines model performance. Particularly for the full model, the general trend was positive interaction between temperature and precipitation (β > 0; Table 1).

**Table 1:** Summary statistics (mean ± SD) of the coefficient of determination (R^2^), Bayesian information criterion (BIC) and the slope coefficient (β coefficient) extracted from the set of models adjusted against the standardized effect size of phylogenetic diversity (SES.PD), mean pairwise distance (SES.MPD) and mean nearest taxon distance (SES.MNTD) against the null model, the additive model of temperature and precipitation (Temp + Temp) and the full model (Temp + Prec + Temp:Prec).

Phylogenetic metric	Model	R^2^	ΔBIC	β coefficient
SES.PD	Null	–	0.20 ± 0.81—**(3.45)**	–
	Temp + Prec	0.20 ± 0.15—**(0.59)**	2.35 ± 1.14—**(0.96)**	Temp.: 0.03 ± 0.09—(**0.25)**, Prec: − 0.02—0.06 (**− 0.32)**
	Temp + Prec + Temp : Prec	0.26 ± 0.15—**(0.79)**	3.7 ± 1.32—**(0)**	Temp: − 0.07 ± 0.12—(− **0.06)**, Prec: − 0.21 ± 0.06—(**− 0.35),** Temp:Prec: 0.09 ± 0.04—(**0.15)**
SES.MPD	Null	–	1.28 ± 0.81—**(3.44)**	–
	Temp + Prec	0.51 ± 0.05—**(0.65)**	0 ± 0—**(0)**	Temp: − 0.04 ± 0.03—(**0.01)**, Prec: − 0.38 ± 0.02—(**− 0.36)**
	Temp + Prec + Temp : Prec	0.56 ± 0.05—**(0.70)**	1.17 ± 0.18—**(0.8)**	Temp.: 0.05 ± 0.02—(**0.10)**, Prec.: − 0.37 ± 0.01—(**− 0.35),** Temp:Prec: − 0.08 ± 0.01—(**− 0.07)**
SES.MNTD	Null	–	0.02 ± 0.2—**(0)**	–
	Temp + Prec	0.07 ± 0.09—**(0.16)**	3.36 ± 0.71—**(2.62)**	Temp: 0.02 ± 0.08—(**0.15)**, Temp: 0.01 ± − 0.08—(**− 0.09)**
	Temp + Prec + Temp : Prec	0.15 ± 0.11—**(0.56)**	4.59 ± 1.09—**(0.02)**	Temp: − 0.09 ± 0.11—(− **0.13)**, Prec: − 0.0004 ± 0.08—(**− 0.13),** Temp:Prec: 0.09 ± 0.06—(**0.24)**

Equally probable models were considered if the difference in BIC (ΔBIC) between the focal model and the model with the lowest BIC were < 2. All parameters were extracted only from models whose residuals met the normality tests. Value obtained from the MCC tree is expressed in the bold number within parenthesis.

### β-Diversity

**Figure 2:**
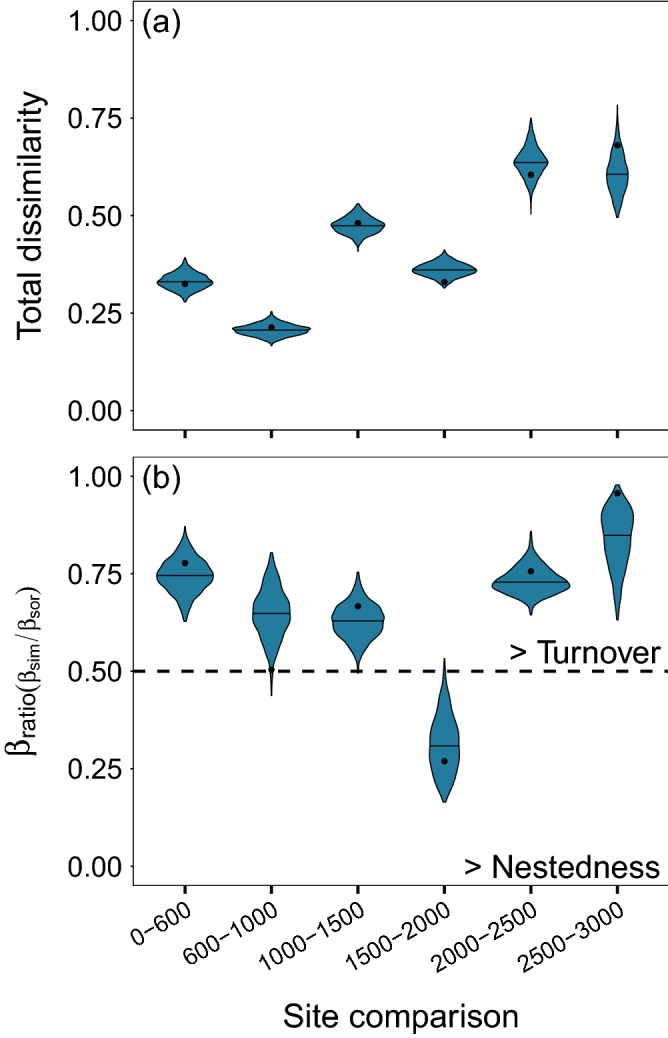
Patterns in the (**a**) phylogenetic total dissimilarity and (**b**) the relative contribution of the turnover over the total dissimilarity (i.e., β_ratio_ = PBD_adj.sim_/PBD_adj.sor_) between adjacent sites along the tropical mountain of Cofre de Perote, Mexico. β_ratio_ > 0.5 indicates that beta diversity is determined dominantly by species turnover, whereas β_ratio_ < 0.5 indicates the predominance of the nestedness component. The inner horizontal line in each violin represents the mean of the 1000 PBD values per site comparison. Black point represents the SES values from the MCC tree.

We found that total phylogenetic dissimilarity derived from multiple-site calculations exhibited considerable high values (PBD_multi.sor_ = 0.73 ± 0.006). Decomposition demonstrated that the turnover component (PBD_multi.sim_ = 0.52 ± 0.02) had a greater contribution to total dissimilarity compared with the relatively low values of the nestedness-resultant component (PBD_multi.nes_ = 0.21 ± 0.01). These patterns remained when the MCC tree was used (Supplementary Fig. S3). When assessing the patterns under the adjacent approach, we observed that the total dissimilarity (PBD_adj.sor_) increased with elevation (e.g., β = 0.08, p-value: 0.04 when values from the MCC tree were regressed with elevation). More specifically, lower values (< 0.5) were observed at lowland sites (< 2000), whereas higher values (> 0.5) at the highlands (> 2000; Fig. 2a). In most cases, the β_ratio_ calculation displayed values higher than 0.5 indicating that total dissimilarity is determined dominantly by turnover. A deviation of this general trend was observed at 1500–2000 sites where the nestedness (PBD_adj.nes_) dominated over the turnover component (Fig. 2b; summary statistics in Supplementary Table S3). Pairwise patterns of PBD showed that an increase of elevational distance results in greater total dissimilarity (PBD_pair.sor_) and is produced by a mixture of turnover (PBD_pair.sim_) and nestedness (PBD_pair.nes_) values operating between different elevational ranges (Supplementary Fig. S4).

According to GDM analysis, the full model explained a considerably high variation of PBD pairwise patterns. Particularly, 79.2% of deviance was explained for total dissimilarity (PBD_pair.sor_), followed by 49.9% for nestedness (PBD_pair.nes_) and in minor instance the turnover (PBD_pair.sim_) with 26.4%. Statistical significance was observed for all total dissimilarity models, however, turnover and nestedness were poorly supported with three and seventy-two (out of 1000) significant models respectively (Table 2). These results are consistent when the MCC tree was used in the analysis (Supplementary Table S4). In all PBD components, the variance explained by the temperature was much larger than explained by geographic distance or precipitation, indicating that thermal affinity has played a much greater role than affinity to precipitation and dispersal limitation in shaping the phylogenetic composition of ant assemblages across our study area (Table 2).

**Table 2:** Summaries of the generalized dissimilarity models adjusted between total phylogenetic dissimilarity (PBDpair.sor), the turnover (PBDpair.sim) and the nestedness-resultant component (PBDpair.nes) against the geographical and climate predictors.

	PBD_pair.sor_	PBD_pair.sim_	PBD_pair.nes_
Model deviance	0.5 (± 0.06)	1.52 (± 0.2)	1.2 (± 0.15)
Deviance explained (%)	79.2 (± 2.5)	26.3 (± 7.6)	49.9 (± 5.1)
P-value ratio	1	0.003	0.072

Predictors	Predictor impact		
Geography	2.2 (± 0.84)	6.6 (± 8.3)	0.28 (± 0.5)
Temperature	59.2 (± 3.07)	48.6 (± 12.4)	66.0 (± 9.0)
Precipitation	23.2 (± 3.8)	20.7 (± 15.8)	18.3 (± 6.5)

All values (except p-value ratio) are represented by the mean of the 1000 phylogenetic trees (± SD). p-value ratio is the proportion of significant models (p < 0.05) out of the 1000 phylogenetic trees.

### Discussion

In this study, we analyzed the phylogenetic α- and β- diversity to unravel the assembly of leaf-litter ant communities along a tropical mountain in Mexico. Overall, lowland and some highland sites show low phylogenetic diversity and are composed of closely-related species (phylogenetic clustering), whereas communities at intermediate elevations and the highest site present higher phylogenetic diversity and are inhabited of distantly-related species (phylogenetic overdispersion). All these patterns were highly supported not only across all the α-phylogenetic metrics (PD, MPD, MNTD) but also across 1000 hypothetical trees and the maximum clade credibility tree, thus patterns remained regardless of phylogenetic uncertainty. We found a positive relationship between temperature interacting with precipitation, suggesting that communities showing high phylogenetic diversity and tending to overdispersion are higher in environments with warm temperatures along with wet and seasonal precipitation regimens. Conversely, communities showing a low phylogenetic diversity and clustering tendency are associated to cold-dry sites with less-seasonal precipitations. Both the additive and full effects of precipitation with temperature were equally supported, yet the full model explained higher variation of phylogenetic α-diversity patterns for all metrics. Phylogenetic total dissimilarity was mainly driven by turnover, thus highland lineages are not subsets of lowland assemblages but instead, they are communities with different lineage compositions. Finally, the temperature differences between sites emerged as the most important driver of total dissimilarity, turnover and nestedness components. In sum, our findings provide additional evidence that evolutionary processes mediated by climate-related (deterministic) mechanisms are strongly involved in the assembly of ant communities along elevational gradients.

Contrary to our expectations, ant communities at the lowland and highland sites (except for the 3000 site) showed a tendency to phylogenetic clustering. Such a pattern has been typically associated with the gradual loss of ant lineages due to abiotic constraints (i.e., environmental filtering) where only a subset of species possesses the necessary adaptations to persist under stressful conditions^25–27^. In our study area, the drastic reduction of temperature coupled with relatively higher precipitation with increasing elevation may act as a strong filter on total diversity and the members of the regional species pool, such that the resultant high-elevation community would be phylogenetically clustered with taxa possessing wet and cold-resistant traits for dealing with this stress. Here, we found that > 50% of species inhabiting phylogenetically clustered communities at high elevations belong mainly to two cold-specialist genera *Stenamma* and *Temnothorax* (Supplementary Table S5). Strategies such as the hibernation of the ant colony during the winter season in *Stenamma* species^44^ or the production of glycerol and other antifreeze substances in *Temnothorax*^45^ may account as likely explanations for the ant persistence in this high, cold environments. Conversely, phylogenetic clustering at lowlands has never been reported for elevational ant studies. The reasons for this deviation require further study, but we propose two likely explanations. First, in comparison with the upper part of the mountain, the lowlands of the Cofre de Perote are characterized by high temperatures and marked precipitation seasonality. This means that all (or almost all) precipitation is concentrated in a relatively short time (3-months), leaving a prolonged drought season for the rest of the year. This drought period interacting with high temperatures may drastically prone ant species to desiccation^46^, and as a result, increase the importance of environmental filtering at those communities^47,48^. The second explanation includes the competition displacement, in which native ants are locally excluded by tramp and alien ant species. The competition displacement may lead to phylogenetic clustering since it is expected that only closely-related taxa of the introduced species can subsist under these biological invasions^24^. In fact, an observable characteristic of our lowland communities is the high dominance of three tramp and alien species: *Hypoponera opaciceps* (Mayr, 1887), *Solenopsis geminata* (Fabricius, 1804) and *Wasmannia auropunctata* (Roger, 1863; Supplementary Table S5). The well-known effects of these ant species in disrupting and displacing native ants^49–51^ suggest that patterns of phylogenetic clustering observed at lowland communities may not be driven by contemporary climate alone, but is also a result of the invasion of these ant species.

We found intermediate elevations tending to phylogenetically overdispersed communities. Typically, phylogenetic overdispersion is interpreted as evidence of interspecific competition since a long history of competitive interactions should cause evolutionary divergence in species niches^52^. Interspecific competition among ant species is intense and often involved in the configuration of ant communities^53^, yet evidence suggests that the importance of competition may be higher in favorable, stable environments where abiotically stressful factors are absent^27,54^. Intermediate elevations at Cofre de Perote reflect these conditions considering that at these elevations temperature is not too low to freeze available water nor too high to evaporate it (Supplementary Fig. S4). Besides, productivity, an ecological proxy of the amount of niches and resource heterogeneity in an ecosystem^55^, is expected to peak at this point since productivity is limited by drought at lowlands and cold temperatures at highlands. Thus, if competition is the driving mechanism at these intermediate elevations, we should observe communities containing a series of species with different evolutionary histories^56^. Indeed, clades are well represented in these communities with seventeen tribes (out of eighteen) containing a mixture of species from both tropical and temperate origins distributed at low and high elevations respectively.

Whilst competitive interaction is congruent as a structuring force in phylogenetically overdispersed communities at more favorable habitats found at intermediate elevations, it is unlikely that this hypothesis stands for the isolated overdispersed community at the highest stressful elevation (i.e., 3000 m). Particularly, we observed that the ant community at this elevation was composed of eight species, each one belonging to different genera dispersed across the phylogeny. Some studies have posited that geographic isolation for historical climatic variations has played a key role in the distribution of species at high-elevation habitats^57–59^. Thus, the presence of species with contrasting evolutionary histories may suggest that communities at 3000 m could be acting as refugia, maintaining relict lineages that migrated from distant regions with temperate climate or were more widespread in the past but became geographically isolated as a consequence of habitat contractions in the last glaciations^58^.

The complementary use of the multiple-site, adjacent, and pairwise approaches, coupled with the decomposition of total dissimilarity into the turnover and nestedness components significantly contributed to unveil the underlying mechanisms influencing dissimilarity variation. For instance, all approaches showed a high dominance of the turnover component for total dissimilarity. This result indicates that the turnover of species is actually a turnover of entire lineages or clades^12^. Besides, it suggests that ant lineages are established at specific elevations (habitat specialization) corresponding to the climatic conditions where they originated^18^. A deeper examination of the distribution of large clades along the Cofre de Perote may support this assumption. On one hand, several ant genera within the tribes Attini (e.g., *Octostruma*, *Pheidole* and *Strumigenys*) and Solenopsidini (e.g., *Solenopsis, Monomorium, Megalomyrmex*) belonging to the subfamily Myrmicinae are highly restricted to the warm conditions found at lowlands probably resembling their neotropical origin^42,60^. On the other hand, middle elevations are highly dominated by *Adelomyrmex* species. This genus is considered as pantropically distributed but its dominance in cloud forests, such as those predominating in our sampled intermediate elevations, is well documented^61,62^. Finally, lineages with more temperate origins such as *Stenamma*^63^ and *Temnothorax*^64^ show a tendency to specialize at more high elevations and rarely spread to lowlands (Supplementary Table S5). Taken together, our results bring evidence that habitat specialization is not the only key driver of compositional dissimilarity of species (i.e.^22,25,32^), but also an important process scaling up to entire lineages (i.e., phylogenetic niche conservatism), in such a way that evolutionary history strongly constraints the elevational distribution of ant species^25^.

Despite the adjacent turnover component having a preponderant impact on total dissimilarity, we observed a breakpoint between 1500 and 2000 where the phylogenetic nestedness prevailed over turnover. The possible reason for this dominant nestedness in PBD is that selective extinction, operating through environmental filtering, is playing an important role in shaping the patterns of phylogenetic dissimilarity at this particular elevation^21^. Because environmental filtering favors certain traits over others, a high number of ant lineages are lost as a result of the temperature decrease and humidity increase occurring from 1500 to 2000 site^65^. Indeed, we noticed that the distribution of certain ant species was restricted to low and intermediate elevations (Supplementary Table S5). Particularly, several well-known neotropical species belonging to *Brachymyrmex*, *Europhalothrix* and *Strumigenys* genera completely disappear at 2000 site (or higher elevation sites; Supplementary Table S5). These ant genera are mainly restricted to more tropical environments found in lowland areas, with one or few species within the same clade capable to cross to high elevations^62^. Therefore, we speculate that this point of the mountain can serve to broadly separate two well-distinguished ant fauna: low-montane fauna, distributed from sea level to below the 2000 m of elevation, and high-montane fauna habiting at 2000 elevation and greater.

We show here that phylogenetic total dissimilarity was best explained by temperature differences among pairwise sites regardless of the precipitation or geographical differences among them. More specifically, higher total dissimilarity is expected between two sites differing in their temperatures in comparison with two sites sharing similar temperatures. This result agrees with those found by Liu et al.^25^ who documented that PBD in ant composition dissimilarity of the Hengduan mountain was mainly driven by climatic distance (in which a set of temperature-related variables were included). This high importance of temperature distance shaping PBD may be explained by the climate variability hypothesis proposed by Janzen^66^. The climate variability hypothesis proposes that species exposed to variable climates (such that occurring at higher elevations) evolve broad thermal tolerances, allowing those species to traverse climatic gradients found across elevations, resulting in a wider geographic distribution than thermal specialists from stable climates (generally found at lower elevations). Therefore, we should expect lineages that originated in more tropical regions (e.g., neotropical) to be characterized by narrower thermal tolerances resulting in more restricted distribution in comparison with lineages originated in more variable climates (e.g., nearctic). The fact that eighty ant species (54%) were found in only one elevation (see Fig. S1 in^32^), many of them restricted to the lowlands and belonging to genera with a neotropical origin (e.g., *Brachymyrmex*, *Camponotus*, *Pheidole, Strumigenys* and *Octostruma*; Supplementary Table S5) supports this assumption. This consistent effect of thermal adaptations constraining not only species distributions^32,67^ but those of entire lineages support that climatic niches are conserved over the evolutionary history of the ant clade^25,43^. Taken together, these results highlight the role of species sorting processes^68^, where the phylogenetic composition is mainly driven by deterministic processes (i.e., habitat filtering) in response to local environmental conditions rather than stochastic processes (i.e., dispersal limitation). The simultaneous examination of the phylogenetic α- and β-diversity (and its components of turnover and nestedness) enhances our understanding of the relative importance of deterministic and stochastic processes in structuring patterns of ant diversity.

Here, we showed that environmental filtering, interspecific competition and habitat specialization jointly structure the leaf-litter ant communities along the Cofre de Perote. These results highlight the importance of deterministic (niche-based) processes over stochastic processes. Further, our results provide insights about phylogenetic niche conservatism since some ant lineages have retained the necessary traits to colonize harsher environments (the colder habitats at the summit). Additionally, the large evolutionary history accumulated in the lineages inhabiting each elevational site along with the remarkable rates of phylogenetic turnover contributing to total phylogenetic dissimilarity confirms the importance of mountains not only as centers of species diversity but crucial reservoirs of unique evolutionary history^69,70^. In concordance with other studies^2,27^, our results highlight the importance of well-focused conservation strategies in mountain systems considering that the increase of anthropogenic influence and global warming threatens the diversity patterns across the tropical mountains. For instance, the expected increase of temperature coupled with the high importance of thermal tolerances in ant species may disrupt lowland communities by forcing species to move up the mountain, leading to losses of distinct evolutionary histories found at the summit due to no possible migration upwards^71^. Altogether, this work builds on the theory that not only contemporary but historical factors also influence the structure of leaf-litter ant assemblages along environmental gradients and this can be detected by integrating α- and β-phylogenetic diversities.

## Methods

### Study area and sant sampling

**Figure 3:**
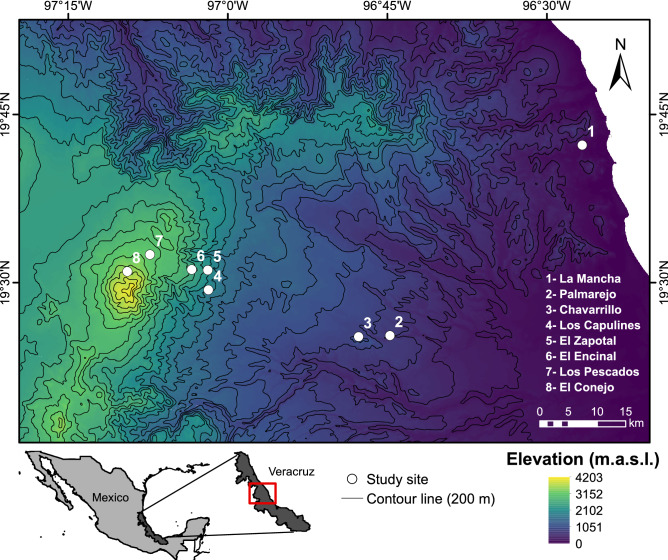
Altitudinal map showing the location of the eight sampling points (white dots and numbers) along the Eastern slope of the Cofre de Perote, Mexico. Elevation data were obtained from INEGI (https://www.inegi.org.mx/app/geo2/elevacionesmex/) and contour lines from CONABIO (https://www.gob.mx/conabio, CONABIO 1998). Map is projected in World Geodetic System of 1984 (WGS84) and was created using ArcGis desktop 10.3, http://www.esri.com/software/arcgis/arcgis-for-desktop.

This study was conducted along the eastern slope of the Cofre de Perote mountain, in Veracruz, Mexico. This region is located at the junction of the Trans-Mexican volcanic belt and the Sierra Madre Oriental. We selected eight study sites spanning an elevational gradient from 0 to 3500 m (Fig. 3). All sites were systematically separated with an elevational difference of 500 m on average between each other. We placed our study sites at the following elevations above sea level: 30–50 m, 610–670 m, 900–1010 m, 1470–1650 m, 2020–2230 m, 2470–2600 m, 3070–3160 m and 3480–3540 m, however, for simplicity, we will refer to each site as discrete units (i.e., 0, 600, 1000, 1500, 2100, 2500, 3100, 3500 m). Geographical distance among adjacent sites varied greatly, ranging from 1.6 km between the closest sites (2000–2500), 44.6 km separating the furthest (0–500) and an average of 14.26 km.

At each sampling point (i.e., elevational site), we collected 40 independent 1 m^2^ forest floor samples separated at least 20 m from each other (i.e., 320 m^2^ across the entire mountain). Ant communities at each elevational site were obtained by pooling all the species from these 40 samples (see^32^ for a complete description of sampling design). In each 1-m^2^ quadrat, we collected the leaf litter inside and sifted it through a coarse mesh screen of 1-cm grid size to remove the largest fragments and concentrate the fine litter. The concentrated fine litter from each sample was suspended in independent mini-Winkler sacks for 3 days in the laboratory. Falling arthropods were collected into a container with 95% ethanol. Ant workers were removed from each container and identified at the species level or assigned to a morphospecies number. Since no ant species were collected at 3500 m, all analyses were conducted excluding this site (see^32^ and Supplementary Table S5).

### Ethics approvals

All applicable international, national and institutional guidelines for the collection of ant specimens and leaf-litter material were followed. All procedures performed in studies involving animals were in accordance with the ethical standards of the institution at which the studies were conducted. The material was collected following the permits issued by SEMARNAT (license number: FAUT-0312).

### Phylogenetic tree constructions

Ideally, one would use a complete, species-level phylogeny of all ant species present in your study area to calculate phylogenetic diversity, yet our current understanding of ant relationships is still limited. As an alternative, we built a genus-level phylogeny based on the tree by Moreau and Bell^72^, but using the phylogenetic relationships and divergence times within Myrmicinae from Ward^42^. This phylogeny was then pruned to keep only a single species per genus to generate a genus-level phylogeny. To maximize taxonomic coverage, we replaced genera that were missing from those studies with closely-related lineages that were not present in our dataset using other phylogenetic studies^73–75^. We then used the list of species (Supplementary Table S5) in our dataset to simulate a species-level phylogeny in which the relationships within genera were obtained from a Yule (pure-birth) process using the *genus.to.species.tree* function in the “phytools” package^76^. A total of 1000 simulated trees were obtained to account for phylogenetic uncertainty (see^77^ and^78^ for similar approach). Additionally, we constructed a Maximum Clade Credibility tree (hereafter MCC tree) which was used to summarize the uncertainty of the 1000 simulated trees. The MCC tree was constructed from the sample of the 1000 trees with the *maxCladeCred* function incorporated in the “ape” package^79^. Both the 1000 hypothetical trees and the MCC tree were used in downstream analyses (Supplementary Fig. S6).

### Alpha and beta phylogenetic diversity

Phylogenetic alpha diversity patterns of leaf-litter ant assemblages at each site were estimated using three metrics: Faith’s PD^80^, mean pairwise distance (MPD) and mean neighbor taxon distance (MNTD). PD measures the total phylogenetic branch length that joins the basal node to the tips of all the species in the sample^80^ and is used as a measure of absolute phylogenetic diversity^81^. MPD and MNTD are two phylogenetic divergence metrics^7,81^, with the former being an estimate of the average phylogenetic relatedness between all possible pairs of taxa in a local community, whereas the latter is an estimate of the mean phylogenetic relatedness between each taxon in a local community and its nearest relatives.

To investigate which processes may be influencing the patterns of phylogenetic alpha diversity, we calculated the standardized effect sizes (SES) for each α-diversity metric (i.e., SES.PD, SES.MPD, SES.MNTD). SES values were calculated by taking the difference between the observed value of PD, MPD and MNTD and their corresponding mean random values derived from null communities, then dividing these differences by the standard deviation across randomizations^82^. The null communities were generated by randomizing the community data matrix using the “independentswap” algorithm with 1000 iterations. Positive and negative SES.PD values indicate species in a community accumulated more or less evolutionary history than expected by null communities, respectively^83^. Whereas for MPD and MNTD, positive SES values suggest phylogenetic overdispersion, whereas clustering is inferred by negative values^7^. Statistical significance is inferred if SES values are greater than 1.96 or less than -1.96. All these analyses were conducted using the *ses.pd*, *ses.mpd* and *ses.mntd* functions incorporated in the “picante” package^84^ of the R software^85^.

We investigated the patterns of β-diversity through three complementary approaches: (i) the multiple-site approach (PBD_multi_), used to summarize in one value the overall dissimilarity in the mountain, (ii) the adjacent approach (PBD_adj_), used to investigate the unidirectional β-diversity variation focusing only on adjacent sites towards the summit, and (iii) the pairwise approach (PBD_pair_), used to investigate how β-diversity patterns were related to the environmental and geographical distance between all pairwise sites. For all approaches, we calculated the total dissimilarity through the PhyloSor distance (multiple-site: PBD_multi.sor_, adjacent: PBD_adj.sor_; pairwise: PBD_pair.sor_) and further decomposed it into the turnover (multiple-site: PBD_multi.sim,_ adjacent: PBD_adj.sim_; pairwise: PBD_pair.sim_) and the nestedness (multiple-site: PBD_multi.nes_, adjacent: PBD_adj.nes_; pairwise: PBD_pair.nes_) components. Under the phylogenetic framework, total dissimilarity captures the proportion of shared and exclusive branch lengths among assemblages, turnover measures ‘true’ lineage turnover and nestedness considers the differences in Faith’s PD between assemblages^21^. Multiple-site calculations were obtained using the *multi.phylo* function, whereas pairwise and adjacent values with the *phylo.beta.pair* function incorporated in the “betapart” package^86^.

To assess the relative contribution of the spatial turnover component to the total dissimilarity between adjacent sites, we calculated the ratio of turnover over total dissimilarity (hereafter β_ratio_) following Dobrovolski et al.^87^: PBD_adj.sim_/ PBD_adj.sor_. Thus, β_ratio_ > 0.5 indicates that total dissimilarity is determined dominantly by the turnover, and β_ratio_ < 0.5 indicates nestedness is the dominant component^87,88^. We did not conduct such analyses for the pairwise approach since the raw-unconverted data is necessary for GDM analyses (see “Statistical analyses” section).

### Climatic predictors

Considering that ants do not respond to elevation directly but rather a suite of covarying abiotic factors^89,90^, we focused in evaluate whether local climate account for the observed phylogenetic diversity patterns. For this purpose we used the 19 climatic variables from the bioclimatic raster available for Mexico at 3 arc-second resolution (~ 90 m^91^) coincident with the twenty georeferenced points at each elevational site. Considering that some sampling points are close enough to prompt to pseudo-replication, only one sampling site was permitted in each predictor raster pixel. This was achieved by excluding duplicated geolocations by using the *gridSample* function in “dismo” package^92^ which retains a single point from each raster pixel. Thus, all values were calculated as the mean of all the independent rasters within each elevational site. We first divided those 19 variables into temperature- and precipitation-related subsets. Then, we used separate principal components analyses (PCA) to generate a synthetic uncorrelated climatic variable that represents the original variables contained in each climatic subset. Before PCA analyses all variables were standardized to remove the unit and were centered (mean = 0, SD = 1). Since the first principal component accounted for a high variation contained in each subset of temperature (PC1_Temperature_:85.3%) and precipitation (PC1_Precipitation_:67.5%; Supplementary Table S6), we conducted the consecutive analyses using only these vectors (hereafter referred simply as “temperature” and “precipitation” respectively). We noted that elevation highly correlated with temperature (ρ = 0.99, p-value < 0.0001) but no with precipitation (ρ = − 0.03, p-value = 0.94).

The examination of variable loading in each principal component revealed that almost all variables included in the analysis (75% of the total) highly contributed (i.e., large weights) to each first component (Supplementary Table S6). Therefore, any interpretation using PC1_Temperature_ and PC1_Precipitation_ should largely reflect the broad variation in terms of temperature and precipitation occurring along the Cofre de Perote mountain.

### Statistical analyses

To evaluate which climatic variables (i.e., temperature and precipitation) better explained the phylogenetic alpha diversity, we implemented multiple regression models where the 1000 SES values of each alpha metric (SES.PD, SES.MPD, SES.MNTD) were regressed with the main effects and the interaction terms of the first principal component of each climatic subset (temperature and precipitation). Simultaneously, we constructed a null model with the same response variables modeled against the intercept. Normality assumption was checked in the residuals of all the adjusted models using the Shapiro test at α = 0.05. To avoid spurious interpretation, a second run of regression models was conducted including only models which met the normality assumption (models syntaxis and number of trees included in final analyses are condensed in Supplementary Table S7).

The null and multiple regression models were evaluated and the model with the lowest Bayesian Information Criterion (BIC) was chosen as the best model^93^. We selected BIC over the Akaike information criterion (AIC) since BIC is based on the assumption that a true model exists among the set of candidate models^94^. We considered this scenario true since temperature and precipitation (and their interaction) have been documented as the most important predictors of ant diversity (e.g.,^30,33,62^). We considered a model equally probable to the best fit model if the difference in BIC (ΔBIC) between the focal model and the model with the lowest BIC were < 2. Further, we extracted the coefficients of determination (R^2^) and the slope coefficient (β) to evaluate the proportion of variance explained by each model and the relationship between each phylogenetic alpha metric with the climatic predictors respectively. Regression models and normality tests were conducted through the *lm* and *shapiro.test* functions respectively, whereas model performance was conducted using the *bictab* function incorporated in the “AICmodavg” package^95^.

To assess whether environmental filtering (climate distances) or dispersal limitation (geographical distances) better explained pairwise PBD patterns, we used Generalized Dissimilarity Modelling (GDM^96^). GDM uses a nonlinear matrix regression technique for analyzing spatial patterns in compositional dissimilarity, providing fitted I-splines to describe the relationships between a dissimilarity matrix (response) and both climatic and geographical predictors, coupled with the partial deviance explained by each predictor^97^. Moreover, GDM standardizes variables so they can be directly compared with one another and is highly robust to multicollinearity among predictors^96^. To conduct GDM, we first converted the observed pairwise dissimilarity matrices (PBD_pair.sor_, PBD_pair.sim_, PBD_pair.nes_) into a GDM site-pair table using the *formatsitepair* function setting the type 3 in the “bioFormat” argument. The *gdm* function was used to fit the model which included the climatic variables (temperature and precipitation) and the geographical coordinates corresponding to the centroid of the total sampling points located at each site. Finally, the function *gdm.varImp* was used to extract the total deviance explained by each model, the significance of the full model and the importance of each predictor. Predictor importance is quantified as the percent change in deviance explained by the full model and the deviance explained by a model fit with that variable permuted^98^. We used 1000 permutations to estimate predictor importance and full model significance. Since this complete procedure was ran across the 1000 matrices of each PDB component, we thus calculated the ratio between the number of significant values (p < 0.05) out of the 1000 phylogenetic trees. GDM analyses were conducted using the functions incorporated in the “gdm” package^98^.

All packages employed for statistical analyses and the “ggplot2” package^99^ used for graph building are incorporated in the R project software (v. 4.1.2)^85^.

## Supplementary Information

Supplementary Information.

## Data Availability

The taxonomic matrix along with the 1000 simulated trees and the Maximum Clade Credibility tree constructed in each elevational site are available on the Zenodo digital repository (https://doi.org/10.5281/zenodo.5646220).
