# Supplementary material for: Alpha and beta phylogenetic diversities jointly reveal ant community assembly mechanisms along a tropical elevational gradient
Source: Sci Rep. 2022 May 11;12:7728. doi: 10.1038/s41598-022-11739-y (PMC9095595; doi:10.1038/s41598-022-11739-y)

SUPPORTING INFORMATION

**Phylogenetic α- and β-diversities jointly reveals leaf-litter ant community assembly mechanisms along a tropical elevational gradient**

**Tables**

**Supplementary Table S1.** Summary statistics calculated for the standardized effect size of phylogenetic diversity (SES.PD), mean pairwise distance (SES.MPD) and mean nearest taxon distance (SES.MNTD). The mean ± SD is provided for the 1000 simulated trees, as well as the unique value calculated for the Maximum Clade Credibility (MCC) tree.

| Sites | SES.PD | |  | SES.MPD | |  | SES.MNTD | |
| --- | --- | --- | --- | --- | --- | --- | --- | --- |
|  | All trees | MCC tree |  | All trees | MCC tree |  | All trees | MCC tree |
| 0 | -0.72 ± 0.46 | -1.34 |  | -0.87 ± 0.07 | -0.86 |  | 0.10 ± 0.54 | -0.61 |
| 600 | -1.50 ± 0.53 | -3.06 |  | -0.53 ± 0.06 | -0.66 |  | -0.72 ± 0.59 | -1.75 |
| 1000 | 0.69 ± 0.50 | 0.65 |  | 0.34 ± 0.06 | 0.28 |  | 0.59 ± 0.57 | 1.29 |
| 1500 | -0.10 ± 0.40 | 0.47 |  | 0.77 ± 0.06 | 0.88 |  | -0.42 ± 0.44 | 0.10 |
| 2000 | -1.26 ± 0.55 | -0.34 |  | 0.25 ± 0.17 | 0.60 |  | -1.56 ± 0.50 | -1.50 |
| 2500 | -2.48 ± 0.66 | -1.02 |  | -3.03 ± 0.45 | -2.13 |  | -1.55 ± 0.52 | -0.48 |
| 3000 | 1.37 ± 0.04 | 1.37 |  | 0.35 ± 0.03 | 0.31 |  | 1.87 ± 0.05 | 1.86 |

**Supplementary Table S2.** Number of phylogenetic trees out of the total (1000) in which the standardized effect size of phylogenetic diversity (SES.PD), mean pairwise distance (SES.MPD) and mean nearest taxon distance (SES.MNTD) laid in the significant statistical area at α=0.05 (< -1.96,> 1.96).

| Sites | SES.PD | |  | SES.MPD | |  | SES.MNTD | |
| --- | --- | --- | --- | --- | --- | --- | --- | --- |
|  | < -1.96 | > 1.96 |  | < -1.96 | > 1.96 |  | < -1.96 | > 1.96 |
| 0 | 1 | - |  | - | - |  | - | - |
| 600 | 202 | - |  | - | - |  | 14 | - |
| 1000 | - | 5 |  | - | - |  | - | 7 |
| 1500 | - | - |  | - | - |  | 1 | - |
| 2000 | 106 | - |  | - | - |  | 209 | - |
| 2500 | 771 | - |  | 995 | - |  | 238 | - |
| 3000 | - | - |  | - | - |  | - | 65 |

**Supplementary Table S3.** Summary statistics calculated for phylogenetic total dissimilarity (PBD_adj.sor_) and the relative contribution of the turnover over the total dissimilarity (β_ratio_= PBD_adj.sim_/PBD_adj.sor_) between adjacent sites going from the base to the summit of the mountain. Information is given in terms of their mean ± SD.

| Site comparison | PBD_adj.sor_ | β_ratio_ |
| --- | --- | --- |
| 0 – 600 | 0.33±0.01 | 0.74±0.04 |
| 600 – 1000 | 0.20±0.01 | 0.64±0.06 |
| 1000 – 1500 | 0.47±0.02 | 0.62±0.04 |
| 1500 -2000 | 0.36±0.01 | 0.31±0.07 |
| 2000 -2500 | 0.63±0.03 | 0.73±0.03 |
| 2500 -3000 | 0.60±0.05 | 0.84±0.07 |

**Supplementary Table S4.** Summary of the generalized dissimilarity models adjusted between total phylogenetic dissimilarity (PBD_pair.sor_)_,_ the turnover (PBD_pair.sim_) and the nestedness-resultant component (PBD_pair.nes_) against the geographical and climate predictors using the maximum clade credibility tree.

|  | PBD_pair.sor_ | PBD_pair.sim_ | PBD_pair.nes_ |
| --- | --- | --- | --- |
| Model deviance | 0.64 | 1.87 | 1.30 |
| Deviance explained (%) | 76.11 | 32.31 | 32.78 |
| P-value | 0.006 | 0.13 | 0.15 |
| Predictors | Predictor impact^a^ |  |  |
| Geography | 3.62 | 18.12 | 0 |
| Temperature | 75.80 | 53.28 | 96.88 |
| Precipitation | 11.08 | 34.01 | 1.46 |

**Supplementary Table S5.** List of species collected in the 320 m^2^ mini-Winkler samples across the eastern slope of Cofre de Perote mountain. The table is filled with species occurrences (i.e., number of 1-m^2^ samples in which a species was collected regardless the number of individuals).

|  | **Site** | | | | | | |
| --- | --- | --- | --- | --- | --- | --- | --- |
| **Species** | **0** | **600** | **1000** | **1500** | **2000** | **2500** | **3000** |
| *Acromyrmex octospinosus* | 0 | 0 | 0 | 1 | 0 | 0 | 0 |
| *Acropyga exsanguis* | 1 | 4 | 10 | 0 | 0 | 0 | 0 |
| *Adelomyrmex dorae* | 0 | 0 | 0 | 6 | 15 | 0 | 0 |
| *Adelomyrmex metzabok* | 0 | 0 | 10 | 0 | 0 | 0 | 0 |
| *Adelomyrmex micans* | 0 | 0 | 0 | 0 | 8 | 0 | 0 |
| *Adelomyrmex nortenyo* | 0 | 0 | 10 | 0 | 0 | 0 | 0 |
| *Adelomyrmex paratristani* | 0 | 0 | 0 | 6 | 0 | 0 | 0 |
| *Adelomyrmex silvestrii* | 0 | 9 | 6 | 0 | 0 | 0 | 0 |
| *Adelomyrmex tristani* | 0 | 0 | 0 | 17 | 1 | 0 | 0 |
| *Anochetus mayri* | 0 | 1 | 0 | 0 | 0 | 0 | 0 |
| *Apterostigma pilosum* | 0 | 2 | 0 | 0 | 0 | 0 | 0 |
| *Belonopelta deletrix* | 0 | 2 | 6 | 0 | 0 | 0 | 0 |
| *Brachymyrmex* sp. 1 | 0 | 1 | 10 | 2 | 0 | 0 | 0 |
| *Brachymyrmex* sp. 2 | 0 | 2 | 0 | 3 | 0 | 0 | 0 |
| *Brachymyrmex* sp. 3 | 0 | 0 | 0 | 3 | 5 | 0 | 0 |
| *Camponotus atriceps* | 1 | 0 | 0 | 0 | 0 | 0 | 0 |
| *Camponotus novogranadensis* | 0 | 1 | 0 | 0 | 0 | 0 | 0 |
| *Camponotus planatus* | 1 | 0 | 0 | 0 | 0 | 0 | 0 |
| *Camponotus striatus* | 0 | 0 | 1 | 0 | 0 | 0 | 0 |
| *Carebara sp. nov* | 0 | 0 | 19 | 0 | 0 | 0 | 0 |
| *Carebara urichi* | 1 | 10 | 5 | 0 | 0 | 0 | 0 |
| *Cephalotes multispinosus* | 0 | 0 | 1 | 0 | 0 | 0 | 0 |
| *Crematogaster cf. coarctata* | 0 | 0 | 0 | 0 | 0 | 1 | 0 |
| *Crematogaster minutissima* | 0 | 1 | 1 | 0 | 0 | 0 | 0 |
| *Crematogaster obscurata* | 1 | 0 | 0 | 0 | 0 | 0 | 0 |
| *Crematogaster saussurei* | 0 | 0 | 0 | 0 | 0 | 0 | 6 |
| *Crematogaster torosa* | 1 | 0 | 0 | 0 | 0 | 0 | 0 |
| *Cryptopone gilva* | 0 | 0 | 0 | 1 | 0 | 0 | 0 |
| *Cyphomyrmex rimosus* | 3 | 10 | 5 | 0 | 0 | 0 | 0 |
| *Discothyrea horni* | 1 | 8 | 11 | 7 | 0 | 0 | 0 |
| *Dolichoderus lutosus* | 0 | 0 | 1 | 0 | 0 | 0 | 0 |
| *Ectatomma ruidum* | 4 | 0 | 0 | 0 | 0 | 0 | 0 |
| *Eurhopalothrix clypeata* | 0 | 0 | 0 | 1 | 0 | 0 | 0 |
| *Eurhopalothrix pilulifera* | 2 | 0 | 0 | 1 | 0 | 0 | 0 |
| *Formica* sp. 1 | 0 | 0 | 0 | 0 | 0 | 0 | 1 |
| *Fulakora orizabana* | 0 | 0 | 0 | 1 | 8 | 0 | 0 |
| *Gnamptogenys hartmani* | 0 | 1 | 1 | 0 | 0 | 0 | 0 |
| *Gnamptogenys minuta* | 0 | 1 | 1 | 0 | 0 | 0 | 0 |
| *Gnamptogenys simulans* | 0 | 0 | 1 | 0 | 0 | 0 | 0 |
| *Gnamptogenys strigata* | 0 | 0 | 0 | 6 | 0 | 0 | 0 |
| *Hypoponera cf. inexorata* | 0 | 0 | 1 | 8 | 0 | 0 | 0 |
| *Hypoponera cf. opacior* | 0 | 1 | 0 | 11 | 24 | 0 | 0 |
| *Hypoponera cf. parva* | 0 | 4 | 17 | 0 | 0 | 0 | 0 |
| *Hypoponera nitidula* | 16 | 7 | 5 | 0 | 0 | 0 | 0 |
| *Hypoponera opaciceps* | 1 | 0 | 0 | 2 | 0 | 0 | 0 |
| *Hypoponera* sp. 1 | 0 | 8 | 10 | 0 | 0 | 0 | 0 |
| *Hypoponera* sp. 2 | 4 | 2 | 0 | 0 | 0 | 0 | 0 |
| *Hypoponera* sp. 3 | 2 | 0 | 0 | 0 | 0 | 0 | 0 |
| *Hypoponera* sp. 4 | 2 | 0 | 0 | 0 | 0 | 0 | 0 |
| *Hypoponera* sp. 5 | 0 | 0 | 1 | 0 | 0 | 0 | 0 |
| *Labidus coecus* | 2 | 0 | 1 | 1 | 0 | 0 | 0 |
| *Megalomyrmex megadrifti* | 0 | 3 | 2 | 0 | 0 | 0 | 0 |
| *Megalomyrmex silvestrii* | 2 | 0 | 0 | 0 | 0 | 0 | 0 |
| *Monomorium ebeninum* | 1 | 0 | 0 | 0 | 0 | 0 | 0 |
| *Monomorium floricola* | 0 | 0 | 0 | 0 | 0 | 0 | 1 |
| *Mycocepurus smithii* | 1 | 1 | 0 | 0 | 0 | 0 | 0 |
| *Myrmelachista skwarrae* | 0 | 0 | 0 | 0 | 1 | 0 | 0 |
| *Myrmicocrypta* sp. 1 | 0 | 2 | 0 | 0 | 0 | 0 | 0 |
| *Neivamyrmex pilosus mexicanus* | 0 | 0 | 0 | 0 | 0 | 0 | 1 |
| *Neivamyrmex swainsonii* | 0 | 0 | 1 | 0 | 0 | 0 | 0 |
| *Nylanderia austroccidua* | 0 | 0 | 0 | 0 | 0 | 1 | 0 |
| *Nylanderia* sp. 1 | 0 | 6 | 1 | 1 | 0 | 0 | 0 |
| *Nylanderia* sp. 2 | 0 | 0 | 0 | 20 | 2 | 0 | 0 |
| *Nylanderia* sp. 3 | 0 | 0 | 0 | 0 | 6 | 0 | 0 |
| *Nylanderia steinheili* | 1 | 10 | 20 | 0 | 1 | 0 | 0 |
| *Octostruma balzani* | 0 | 15 | 27 | 0 | 0 | 0 | 0 |
| *Octostruma planities* | 0 | 1 | 0 | 0 | 0 | 0 | 0 |
| *Octostruma rugiferoides* | 0 | 1 | 0 | 0 | 0 | 0 | 0 |
| *Octostruma trithrix* | 0 | 6 | 6 | 0 | 0 | 0 | 0 |
| *Octostruma wheeleri* | 0 | 8 | 0 | 0 | 0 | 0 | 0 |
| *Odontomachus chelifer* | 0 | 2 | 0 | 0 | 0 | 0 | 0 |
| *Odontomachus laticeps* | 0 | 1 | 0 | 0 | 0 | 0 | 0 |
| *Odontomachus meinerti* | 0 | 1 | 1 | 0 | 0 | 0 | 0 |
| *Pachycondyla harpax* | 11 | 8 | 11 | 0 | 0 | 0 | 0 |
| *Paratrachymyrmex intermedius* | 1 | 0 | 0 | 0 | 0 | 0 | 0 |
| *Pheidole agricola* | 1 | 0 | 0 | 0 | 0 | 0 | 0 |
| *Pheidole albipes* | 0 | 9 | 12 | 0 | 0 | 0 | 0 |
| *Pheidole bilimeki* | 0 | 2 | 2 | 0 | 0 | 0 | 0 |
| *Pheidole ceres* | 0 | 0 | 0 | 0 | 0 | 0 | 3 |
| *Pheidole fimbriata* | 0 | 3 | 2 | 0 | 0 | 0 | 0 |
| *Pheidole glomericeps* | 0 | 0 | 10 | 0 | 0 | 0 | 0 |
| *Pheidole harrisonfordi* | 9 | 11 | 10 | 0 | 0 | 0 | 0 |
| *Pheidole lamancha* | 1 | 0 | 0 | 0 | 0 | 0 | 0 |
| *Pheidole prattorum* | 0 | 0 | 0 | 1 | 0 | 0 | 0 |
| *Pheidole punctatissima* | 0 | 0 | 2 | 0 | 0 | 0 | 0 |
| *Pheidole rectispina* | 0 | 0 | 7 | 0 | 0 | 0 | 0 |
| *Pheidole roushae* | 0 | 0 | 1 | 0 | 0 | 0 | 0 |
| *Pheidole simonsi* | 0 | 2 | 0 | 0 | 0 | 0 | 0 |
| *Pheidole susannae* | 0 | 1 | 0 | 0 | 0 | 0 | 0 |
| *Pheidole tschinkeli* | 0 | 4 | 3 | 0 | 0 | 0 | 0 |
| *Ponera exotica* | 0 | 0 | 0 | 28 | 3 | 0 | 0 |
| *Prionopelta modesta* | 2 | 12 | 1 | 0 | 0 | 0 | 1 |
| *Proceratium mancum* | 0 | 0 | 10 | 0 | 0 | 0 | 0 |
| *Pseudomyrmex boopis* | 1 | 1 | 2 | 0 | 0 | 0 | 0 |
| *Pseudomyrmex oculatus* | 0 | 1 | 0 | 0 | 0 | 0 | 0 |
| *Rasopone ferruginea* | 0 | 2 | 2 | 0 | 0 | 0 | 0 |
| *Rhopalothrix isthmica* | 0 | 0 | 1 | 0 | 0 | 0 | 0 |
| *Rhopalothrix triumphalis* | 0 | 2 | 1 | 0 | 0 | 0 | 0 |
| *Rogeria belti* | 0 | 0 | 0 | 1 | 0 | 0 | 0 |
| *Rogeria cornuta* | 0 | 8 | 5 | 0 | 0 | 0 | 0 |
| *Rogeria cuneola* | 4 | 3 | 4 | 0 | 0 | 0 | 0 |
| *Rogeria innotabilis* | 4 | 0 | 0 | 0 | 0 | 0 | 0 |
| *Solenopsis brevicornis* | 9 | 10 | 15 | 0 | 0 | 0 | 0 |
| *Solenopsis cf. striata* | 0 | 0 | 2 | 0 | 0 | 0 | 0 |
| *Solenopsis geminata* | 16 | 0 | 4 | 2 | 0 | 0 | 0 |
| *Solenopsis pygmaea* | 5 | 0 | 0 | 0 | 0 | 0 | 0 |
| *Solenopsis* sp. 1 | 18 | 17 | 28 | 0 | 0 | 0 | 0 |
| *Solenopsis stricta* | 3 | 2 | 0 | 0 | 0 | 0 | 0 |
| *Solenopsis vinsoni* | 1 | 0 | 0 | 0 | 0 | 0 | 0 |
| *Stenamma connectum* | 0 | 0 | 0 | 31 | 6 | 0 | 0 |
| *Stenamma excisum* | 0 | 0 | 0 | 6 | 0 | 0 | 0 |
| *Stenamma huachucanum* | 0 | 0 | 0 | 0 | 12 | 5 | 0 |
| *Stenamma lobinodus* | 0 | 1 | 13 | 0 | 0 | 0 | 0 |
| *Stenamma manni* | 0 | 0 | 0 | 0 | 0 | 1 | 0 |
| *Stenamma maximon* | 0 | 0 | 0 | 0 | 0 | 19 | 0 |
| *Stenamma pelophilum* | 0 | 0 | 1 | 2 | 3 | 0 | 0 |
| *Stenamma* sp. 1 | 0 | 0 | 0 | 0 | 0 | 0 | 2 |
| *Stenamma stictosomum* | 0 | 0 | 0 | 1 | 3 | 1 | 0 |
| *Stenamma vexator* | 0 | 0 | 0 | 32 | 24 | 16 | 0 |
| *Strumigenys boneti* | 5 | 0 | 0 | 0 | 0 | 0 | 0 |
| *Strumigenys brevicornis* | 1 | 3 | 17 | 22 | 0 | 1 | 0 |
| *Strumigenys cordovensis* | 0 | 0 | 1 | 0 | 0 | 0 | 0 |
| *Strumigenys eggersi* | 11 | 0 | 0 | 0 | 0 | 0 | 0 |
| *Strumigenys elongata* | 1 | 2 | 9 | 0 | 0 | 0 | 0 |
| *Strumigenys emeryi* | 0 | 1 | 0 | 0 | 0 | 0 | 0 |
| *Strumigenys gundlachi* | 0 | 9 | 22 | 0 | 0 | 0 | 0 |
| *Strumigenys lanuginosa* | 0 | 3 | 0 | 0 | 0 | 0 | 0 |
| *Strumigenys louisinae* | 1 | 0 | 0 | 0 | 0 | 0 | 0 |
| *Strumigenys ludia* | 4 | 13 | 9 | 0 | 0 | 0 | 0 |
| *Strumigenys margaritae* | 0 | 1 | 2 | 0 | 0 | 0 | 0 |
| *Strumigenys prex* | 0 | 1 | 3 | 1 | 0 | 0 | 0 |
| *Strumigenys sp. nov* | 0 | 0 | 1 | 0 | 0 | 0 | 0 |
| *Strumigenys spathula* | 0 | 1 | 0 | 0 | 0 | 0 | 0 |
| *Strumigenys subedentata* | 0 | 2 | 1 | 0 | 0 | 0 | 0 |
| *Syscia augustae* | 0 | 0 | 4 | 0 | 0 | 0 | 0 |
| *Syscia* sp. 1 | 0 | 0 | 2 | 0 | 0 | 0 | 0 |
| *Syscia tolteca* | 0 | 0 | 2 | 4 | 4 | 0 | 0 |
| *Tatuidris tatusia* | 0 | 1 | 14 | 0 | 0 | 0 | 0 |
| *Temnothorax* sp. 1 | 0 | 0 | 8 | 0 | 0 | 0 | 0 |
| *Temnothorax* sp. 2 | 0 | 0 | 1 | 0 | 0 | 0 | 0 |
| *Temnothorax* sp. 3 | 0 | 0 | 0 | 1 | 0 | 0 | 0 |
| *Temnothorax* sp. 4 | 0 | 0 | 0 | 0 | 0 | 0 | 5 |
| *Temnothorax sp. nov* | 0 | 0 | 0 | 12 | 0 | 0 | 0 |
| *Temnothorax striatulus* | 0 | 0 | 0 | 0 | 0 | 1 | 0 |
| *Wasmannia auropunctata* | 4 | 16 | 30 | 0 | 0 | 0 | 0 |

**Supplementary Table S6.** Synthetic variables obtained through principal component analyses for both temperature and precipitation related variables of bioclimatic rasters obtained from Cuervo-Robayo et al., 2014. Each first principal component (PC1) expresses the bioclimatic variable included in the PCA, the total variance explanation and the vectors for each of the seven elevational sites at the Cofre de Perote, Mexico. Finally, the loadings for each variable incorporated in PCA is given. All values are standardized (mean=0, sd=1) to remove the original scale and be comparable between each other.

|  | PC1_Temperature_ | PC1_Precipitation_ |
| --- | --- | --- |
| Bioclimatic variables | 1-11 | 12-19 |
| Explained variance (%) | 85.3 | 67.5 |
| Eigenvalues | 3.05 | 2.38 |
| Elevational sites | | |
| 0 | -3.89 | 1.59 |
| 600 | -2.98 | 1.98 |
| 1000 | -2.05 | -1.39 |
| 1500 | 0.31 | -4.07 |
| 2000 | 1.74 | -1.54 |
| 2500 | 2.69 | 2.32 |
| 3000 | 4.17 | 1.10 |
| Loadings |  |  |
| BIO1 | -0.32 | - |
| BIO2 | 0.15 | - |
| BIO3 | 0.31 | - |
| BIO4 | -0.32 | - |
| BIO5 | -0.32 | - |
| BIO6 | -0.32 | - |
| BIO7 | -0.17 | - |
| BIO8 | -0.32 | - |
| BIO9 | -0.32 | **-** |
| BIO10 | -0.32 | **-** |
| BIO11 | -0.32 | **-** |
| BIO12 | - | -0.40 |
| BIO13 | - | -0.37 |
| BIO14 | - | -0.39 |
| BIO15 | - | 0.22 |
| BIO16 | - | -0.34 |
| BIO17 | - | -0.39 |
| BIO18 | - | -0.20 |
| BIO19 | - | -0.39 |

**Supplementary Table S7.** Summaries of simple regression (SR) and multiple regression (MR) models fitting standardized effect sizes of phylogenetic diversity (SES.PD), mean pairwise distance (SES.MPD), and mean nearest taxon distance (SES.MNTD) against the null model (intercept only), the additive model with principal component of temperature and precipitation (Temp + Prec) and the full model (Temp + Prec + Temp:Prec). After each model, it is specified how many trees out of 1000 were included in the final analysis after removing non-normal models after a first running (see Methods in main text).

| Regression model | Type | No. of phylogenetic trees included |
| --- | --- | --- |
| SES.PD ~ Intercept | SR | 1000 |
| SES.PD ~ Temp + Prec | MR | 998 |
| SES.PD ~ Temp + Prec + Temp:Prec | MR | 1000 |
| SES.MPD ~ Intercept | SR | 1000 |
| SES.MPD ~ Temp + Prec | MR | 1000 |
| SES.MPD ~ Temp + Prec + Temp:Prec | MR | 1000 |
| SES.MNTD ~ Intercept | SR | 1000 |
| SES.MNTD ~ Temp + Prec | MR | 998 |
| SES.MNTD ~ Temp + Prec + Temp:Prec | MR | 1000 |

**Figures**

**Supplementary Figure S1.** Distribution of the coefficient of determination (R^2^) of the linear relationship between SES.PD (left), SES.MPD (middle) and SES.MNTD (right) with the additive effect of temperature and precipitation (top) and the full model (bottom) . For each plot is represented the value from the consensus tree (red line) and the mean out of the 1000 phylogenetic trees analyses (black line).


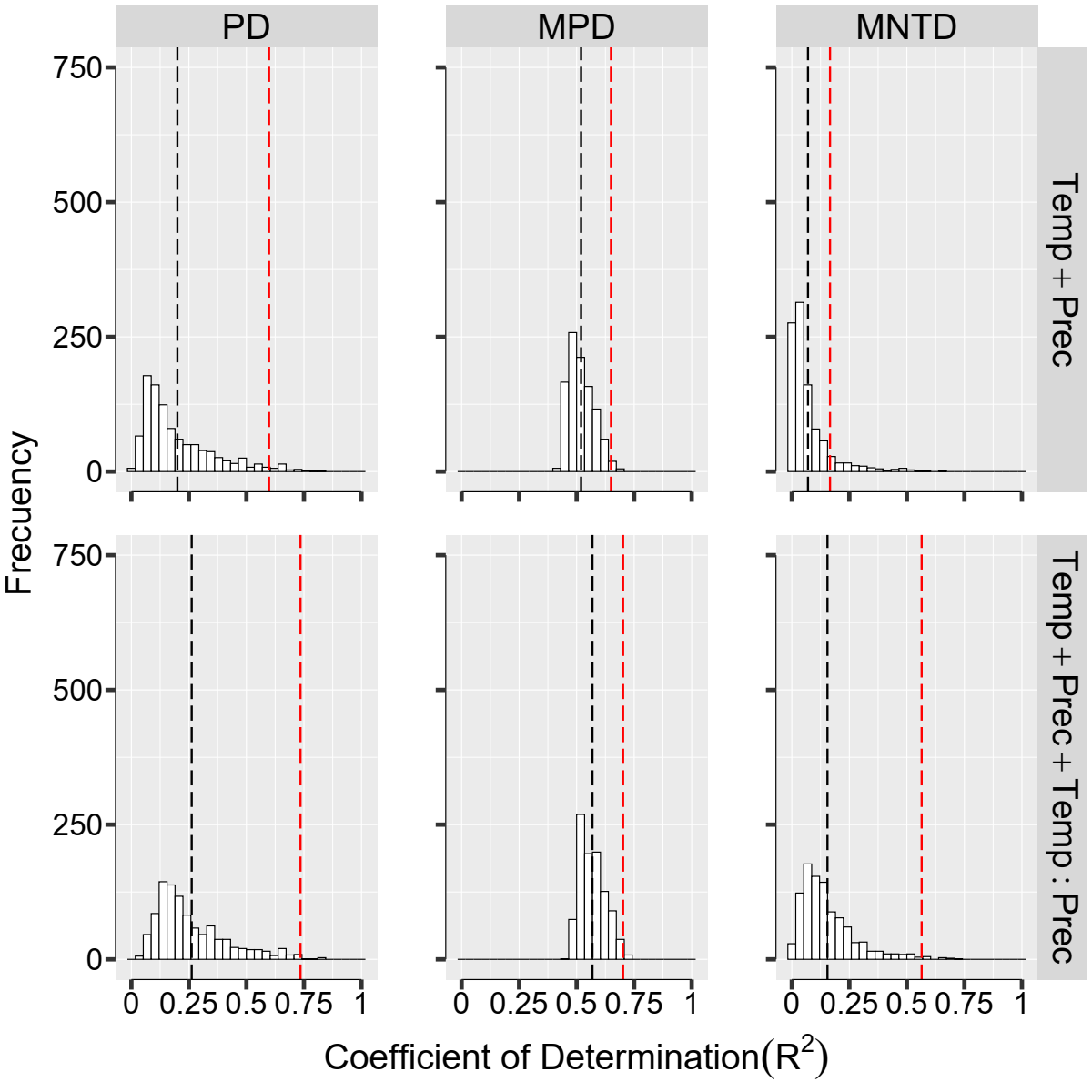


**Supplementary Figure S2.** Distribution of delta Bayesian information criterion (ΔBIC) of the linear regression between the (A) phylogenetic diversity (PD); (B) mean pairwise distance (MPD) and (C) the mean nearest taxon distance against null model, the additive model of temperature and precipitation (Temp + Prec) and the full model (Temp + Prec + Temp:Prec). Models with ΔBIC >2 were considered as equally probable models (area below the solid horizontal line).


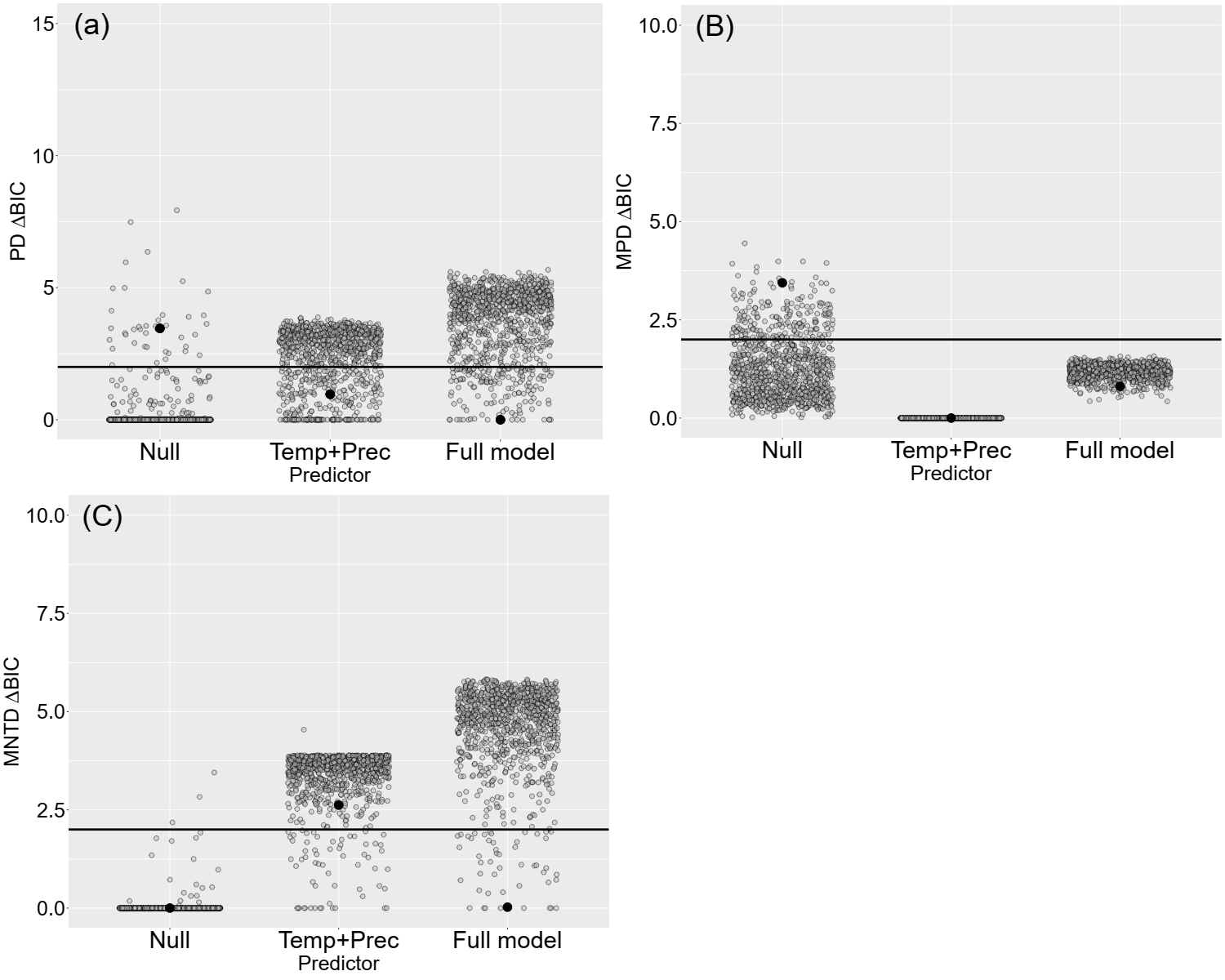


**Supplementary Figure S3.** Multi-site patterns of phylogenetic beta diversity along the Cofre de Perote mountain. Violins represents the 1000 values calculated for each metric: β_SOR:_ Total phylogenetic dissimilarity, β_SIM:_ Phylogenetic turnover and β_NES:_ Phylogenetic nestedness. The additive effect of β_NES_ and β_SIM_ equals β_SOR_. The mean of the 1000 values is represented as the horizontal line within the violin, whereas the value calculated using the maximum clade credibility tree as the black dot.


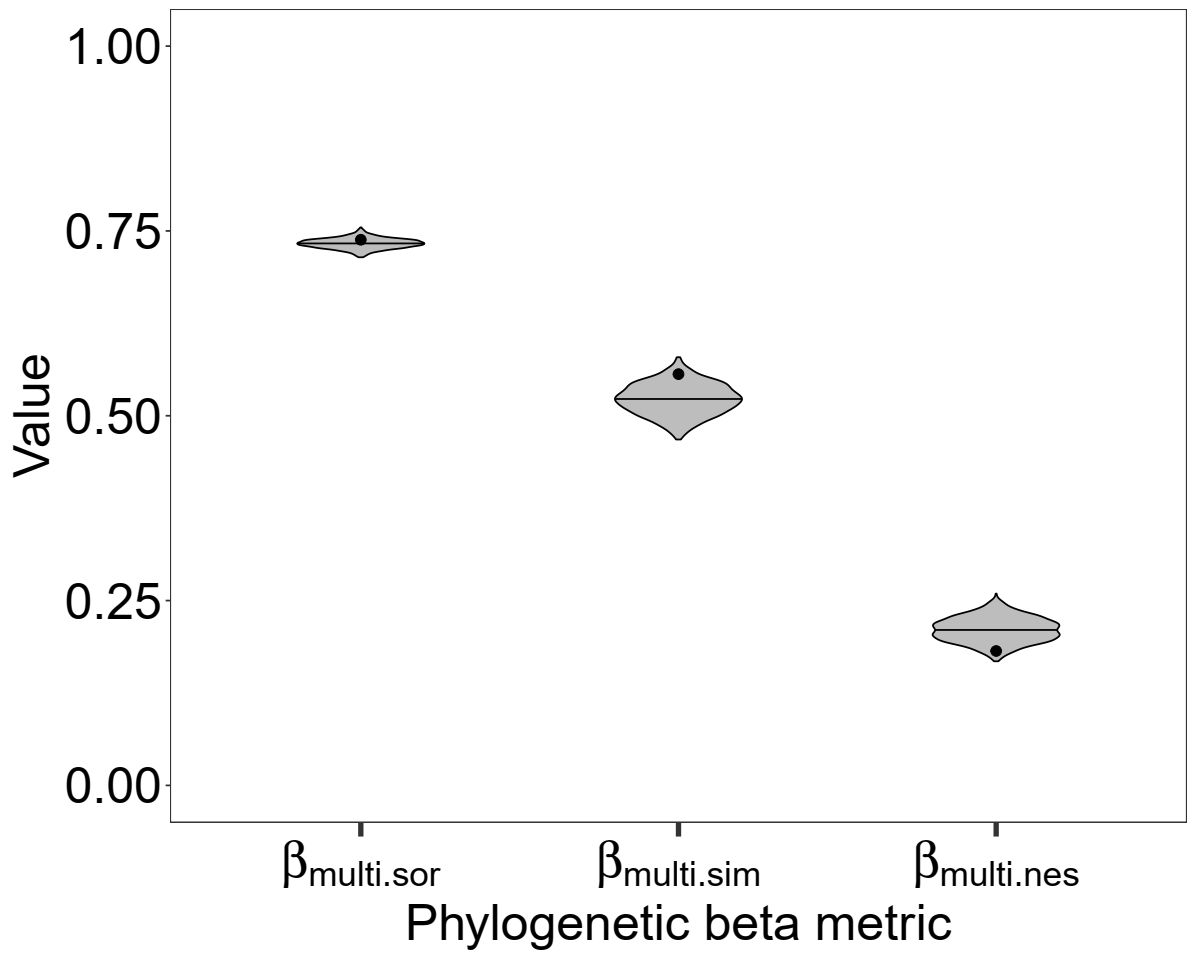


**Supplementary Figure S4.** Relationship between the of a) PBD_pair.sor_= phylogenetic total dissimilarity, b) PBD_pair.sim_=phylogenetic turnover, and c) PBD_pair.nes_= the nestedness component and elevational distance in the Cofre de Perote. The sum of PBD_pair.sim_ and PBD_pair.nes_ equals PBD_pair.sor_. Each gray violins contains the distribution of the 1000 values calculated for each PBD metric (PBD_pair.sor_, PBD_pair.sim_ and PBD_pair.nes_). Black dots represent the mean at each possible elevational difference combination.


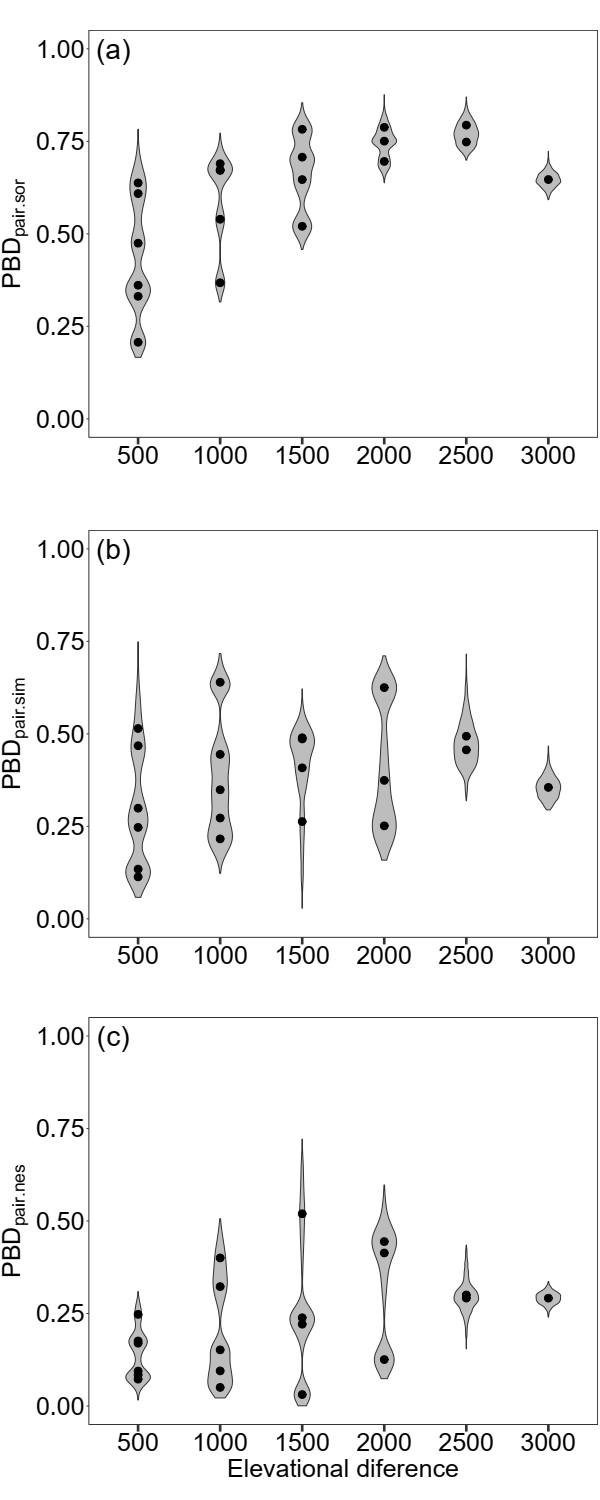


**Supplementary Figure S5.** Climatic characteristics of the seven elevational sites using the 19 WorldClim variables along the study sites of the Cofre de Perote mountain. All values were scaled to be comparable between each other. **BIO1** = Mean Annual Temperature, **BIO2** = Mean Diurnal Range (Mean of monthly (max t° - min t°)), **BIO3** = Isothermality (BIO2/BIO7) (×100), **BIO4** = Temperature Seasonality (standard deviation ×100), **BIO5** = Max Temperature of Warmest Month, **BIO6** = Min Temperature of Coldest Month, **BIO7** = Temperature Annual Range (BIO5-BIO6), **BIO8** = Mean Temperature of Wettest Quarter, **BIO9** = Mean Temperature of Driest Quarter, **BIO10** = Mean Temperature of Warmest Quarter, **BIO11** = Mean Temperature of Coldest Quarter, **BIO12** = Annual Precipitation, **BIO13** = Precipitation of Wettest Month, **BIO14** = Precipitation of Driest Month, **BIO15** = Precipitation Seasonality (Coefficient of Variation), **BIO16** = Precipitation of Wettest Quarter, **BIO17** = Precipitation of Driest Quarter, **BIO18** = Precipitation of Warmest Quarter, **BIO19** = Precipitation of Coldest Quarter.


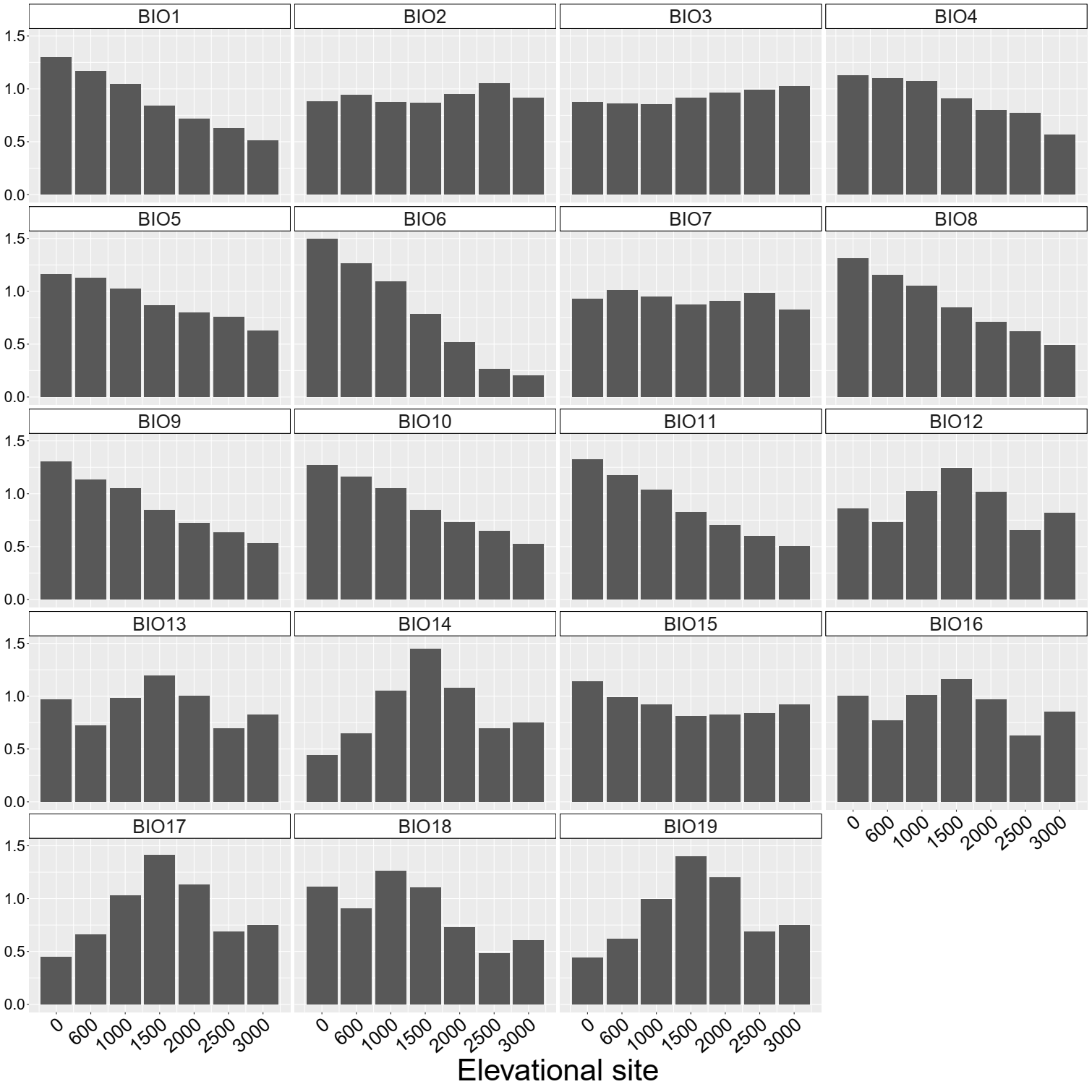


**Supplementary Figure S6**.- Analytical pathway followed to analyze both phylogenetic α- and β-diversity of leaf-litter ant communities along the elevational gradient in the Cofre de Perote, Mexico.


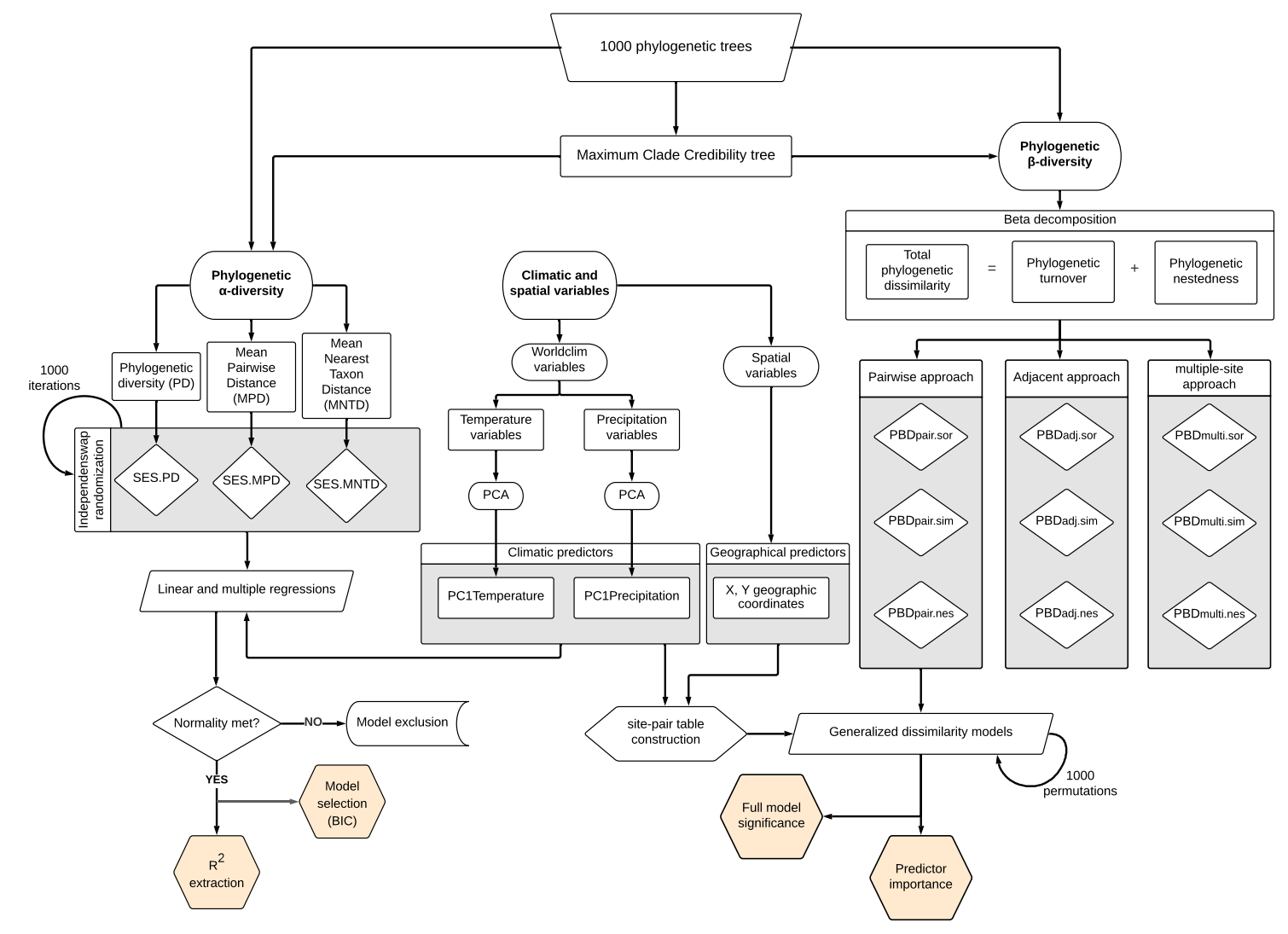

Supplement: Supplementary file 1 — Supplementary Information. [file 41598_2022_11739_MOESM1_ESM.docx]
